# Antibiotic dosing in obesity: a BIG challenge

**DOI:** 10.1186/s13054-016-1426-y

**Published:** 2016-08-10

**Authors:** Timothy P. Hanrahan, Jeffrey Lipman, Jason A. Roberts

**Affiliations:** 1 Burns Trauma and Critical Care Research Centre, The University of Queensland, Brisbane, Queensland Australia; 2 Department of Intensive Care Medicine, The Royal Brisbane and Women’s Hospital, Brisbane, Queensland Australia; 3 Pharmacy Department, The Royal Brisbane and Women’s Hospital, Brisbane, Queensland Australia; 4 School of Pharmacy, The University of Queensland, Brisbane, Queensland Australia

When almost 60 % of the world’s population is predicted to be overweight by 2030 [1], dosing regimens that are developed and tested in non-obese patients will be inappropriate for the future use of drugs. Specifically, should we no longer accept the “one-size-fits-all” mentality of antibiotic dosing and accept that *individuals* may in fact need to be dosed… *individually*? Almost all clinicians use antibiotics daily. Most use guidelines as dosing rules rather than a guide. Particularly in critical illness where dramatic changes in antibiotic concentrations can occur with similar doses, accepting guidelines as dosing rules is likely to be flawed [2].

Antibiotic pharmacokinetics are different in the critically ill compared with other patient groups [3]. Firstly, there are changes to the volume of distribution (Vd). Particularly in patients with sepsis, fluid shifts from the intravascular space to the interstitium lowers the intravascular concentrations of hydrophilic antimicrobials [4]. Furthermore, given a decrease in plasma albumin concentration is seen in approximately 40 % of the critically ill, antimicrobials may further extravasate, additionally increasing Vd. Furthermore, drug clearance may be altered in those with renal impairment (hydrophilic drugs) or hepatic impairment (largely lipophilic drugs). Moreover, there is increasing evidence of enhanced renal elimination of renally cleared drugs (augmented renal clearance) in the critically ill [5].

In the obese, there can be a significant change in the Vd of both hydrophilic and lipophilic antimicrobials consequential to increases in both adipose and lean muscle mass. The degree to which Vd is altered is generally regarded to be a function of the lipophillicity of the drug, although hydrophilic antimicrobials also have Vd alterations secondary to an increased volume of lean muscle, the significance of which is debated [6].

Additionally, the precise effect obesity has on antimicrobial clearance is unclear, with literature scarce. In healthy obese patients renal flow is augmented compared with non-obese [7]. The exact mechanism is debated, though “obesity-related glomerulopathy”, a collective term for glomerulomegaly, with or without focal segmental glomerulosclerosis, along with increased renal plasma flow and associated increased glomerular filtration rate is likely to be the cause [8]. As patients age and obesity-related nephropathies develop, however, renal function can be reduced [9]. Subsequently, obese patients may develop reduced drug clearance compared with age-matched comparators, especially those critically ill with augmented renal clearance.

The abovementioned changes to both Vd and clearance become especially important in those patients that are “super obese”. Significantly higher loading doses are likely to be necessary to accommodate the increased Vd, whilst comparatively lower maintenance doses may be required to avoid drug toxicities in those with reduced clearance. Clearly, when combining the effects of being critically ill and obese, it is difficult to accurately predict the pharmacokinetics of any given antimicrobial. Ultimately, the assumption of linear correlations between lean or total body weight, Vd and drug clearance is problematic and prospective pharmacokinetic trials in critically ill obese patients should be performed to define robust dosing guidelines.

Vancomycin is a glycopeptide antibiotic whose clinical response is dependent on the 24-h area-under-the-concentration-time curve (AUC) to minimum inhibitory concentration (MIC) ratio. It is generally accepted that a target AUC:MIC ratio >400 is optimal [10]. Despite being hydrophilic, vancomycin has a wide Vd in critically ill patients (>1.0 L/kg) and >90 % is renally cleared. As such, taking into account the abovementioned pharmacokinetic alterations in both the obese and the critically ill, dosing can be challenging.

Recent literature comparing vancomycin dosing requirements administered by continuous infusion in obese versus non-obese patients has presented two interesting findings. Firstly, the daily weight-based vancomycin dose was significantly lower for obese compared with non-obese patients when administered by continuous infusion whilst achieving target concentrations [11]. This is unsurprising given clearance is the primary determinant of maintaining steady state concentrations after initial loading is complete [12]. Furthermore, no significant difference in non-weight normalized vancomycin clearance between obese and non-obese patients with preserved renal function was found with minimal correlation to total body weight [11]. This implies that measures of renal function rather than measures of weight are most important for maintenance dosing. These data are similar to recent findings in non-obese critically ill populations [12], although there are ultimately many ill-defined variables that preclude direct extrapolation of non-obese data.

Ultimately, the paucity of data in obese, critically ill populations may force us to accept that therapeutic drug monitoring (TDM) is still the way forward. There is, however, a caveat: the AUC:MIC ratio is rarely measured in clinical practice. Rather, serum trough concentration targets of 15–20 mg/L are advocated as a reliable surrogate. Neely et al. [13] recently showed, however, that the AUC was underestimated by an average of 23 % when using trough concentrations alone, leading to potential excessive vancomycin exposure and unnecessary risks of toxicity. As such, caution when relying on this alone is prudent.

Whilst vancomycin concentrations are easily measured, concentrations of other antibiotics used in obese patients are measured far less frequently. For example, a recent review [14] concluded that very few hospitals worldwide perform beta-lactam TDM on a routine basis despite its availability likely being much higher [15]. This disparity may be because, traditionally, beta-lactam antibiotics had a wide therapeutic window and risk of toxicity was low (compared with other antibiotics routinely measured). However, given the effectiveness of beta-lactam antibiotics depends on the duration that serum concentrations are greater than the target pathogen’s MIC and there is increasing evidence of beta-lactam resistance, it is easy to see that we may inadvertently dose patients poorly if assumptions of uniform pharmacokinetics between non-obese and obese populations are made.

In conclusion, vancomycin clearance may be similar in obese and non-obese populations, with smaller weight-based dosages required to maintain steady state concentrations. Despite this, given the relative paucity of prospective data surrounding antibiotic pharmacokinetics in the combined obese and critically ill cohort, it is clear that TDM makes sense to ensure target concentrations are achieved to increase the likelihood of clinical efficacy.

## Abbreviations

AUC, area-under-the-concentration-time curve; MIC, minimum inhibitory concentration; TDM, therapeutic drug monitoring; Vd, volume of distribution
